# Mitochondrial tRNA methylation in Alzheimer’s disease and progressive supranuclear palsy

**DOI:** 10.1186/s12920-020-0727-9

**Published:** 2020-05-19

**Authors:** Talisa K. Silzer, Gita A. Pathak, Nicole R. Phillips

**Affiliations:** Department of Microbiology, Immunology and Genetics; Graduate School of Biomedical Science, University of North Texas Health Science Center, 3500 Camp Bowie Blvd., Fort Worth, TX USA

**Keywords:** Mitochondria, Methylation, Neurodegenerative disease, tRNA

## Abstract

**Background:**

Methylation of mitochondrial tRNAs (mt-tRNA) at the 9th position (“p9 site”) is known to impact translational efficiency and downstream mitochondrial function; however, direct assessment of mt-RNA methylation is challenging. Recent RNA sequence-based methods have been developed to reliably identify post-transcriptional methylation. Though p9 methylation has been studied in healthy human populations and in the context of cancer, it has not yet been analyzed in neurodegenerative disease, where mitochondrial dysfunction is a prominent and early hallmark of disease progression.

**Methods:**

Mitochondrial p9 methylation was inferred from multi-allelic calls in RNA-seq data. Gene-based association studies were performed in FUMA. Correlations between nuclear gene expression and p9 methylation were tested using Spearman’s rho. Fisher’s Exact test was used in PANTHER and IPA to test for overrepresentation and enrichment of biological processes and pathways in the top nuclear genes correlated with p9 methylation.

**Results:**

Variable methylation was observed at 11 p9 sites in post-mortem cerebellar tissue of elderly subjects who were either healthy or diagnosed with Alzheimer’s disease (AD), progressive supranuclear palsy (PSP) or pathological aging (PA). Similarities in degree of methylation were observed between AD and PSP. Certain nuclear encoded genes were identified as significantly associated with p9 methylation. Expression of 5300 nuclear encoded genes was significantly correlated with p9 methylation, with AD and PSP subjects exhibiting similar expression profiles. Overrepresentation and enrichment testing using the top transcripts revealed enrichment for a number of molecular processes, terms and pathways including many of which that were mitochondrial-related.

**Conclusion:**

With mitochondrial dysfunction being an established hallmark of neurodegenerative disease pathophysiology, this work sheds light on the potential molecular underpinnings of this dysfunction. Here we show overlap in cerebellar pathophysiology between common tauopathies such as Alzheimer’s disease and progressive supranuclear palsy. Whether p9 hypermethylation is a cause or consequence of pathology remains an area of focus.

## Background

Mitochondrial organelles play essential roles in oxidative phosphorylation, apoptosis, calcium homeostasis and a number of cell-signalling pathways. Many lines of evidence have implicated mitochondrial dysfunction in aging and neurodegenerative disease. Though the genetic etiology of neurodegeneration is highly heterogeneous, there is an extensive literature implicating mitochondrial dysfunction as a common and early factor in the pathophysiology of a number of neurodegenerative diseases [1]. Much of the mitochondrial genetic-centric studies of neurodegenerative diseases and related comorbidities have focused primarily on mitochondrial DNA (mtDNA) mutations, changes to mtDNA copy number and/or release of cell-free mtDNA [2]; however, the significance of mitochondrial transcriptome epigenetics and altered mitochondrial RNA (mt-RNA) processing has not been explored. As post-transcriptional processing can alter mitochondrial protein synthesis, aberrant modifications to mitochondrial tRNAs (mt-tRNA) may contribute to the mitochondrial dysfunction underlying neurodegeneration and other age-related pathologies.

Mitochondria contain their own 16,569 base pair genome consisting of 13 mRNA genes encoding electron transport chain subunits and 2 rRNA genes, which are ‘punctuated’ by 22 tRNA genes [3]. The interspersed tRNA sequences serve as important sites for post-transcriptional processing of the polycistronic transcript, accomplished in part by key modifications at several sites throughout the tRNA structure [4]. One of the most common modification types is methylation of adenosine or guanosine residues [4]. Of particular interest is methylation occurring at the 9th position (termed as “p9 sites” here) of mt-tRNAs, as this modification is known to be essential for proper tRNA folding, stability, and decoding capacity [5–8]. Within mt-tRNAs, methyl groups are added by mitochondrial ribonuclease P protein 1 (MRPP1) to adenosine to create 1-methyladenosine (m1A). Lack of modification at this position has been associated with rare neurodegenerative disorders such as HSD10 disease [9]. Further, knockdown of key processing molecules such as MRPP1 has been shown to lead to accumulation of improperly processed tRNAs resulting in decreased mitochondrial protein synthesis [10].

Though various methods exist for measuring RNA methylation including in situ hybridization, methylation-specific antibodies and methylation-sensitive enzymes, many methods lack specificity and/or have limited experimental use, due to the high concentration of input RNA required [11]. Several sequence-based protocols have been established and validated demonstrating a strong correlation between variation in mt-RNA sequence data and degree of post-transcriptional methylation of mt-tRNA at specific sites [10, 12, 13]. In brief, the extent of multiallelic calls (seemingly heteroplasmy) within mt-RNA sequence reads can be used to infer degree of post-transcriptional methylation based on the assumption that chemical modifications such as methyl groups, block reverse transcriptase during cDNA synthesis, often leading to incorporation of incorrect nucleotides [14]. Though useful for detecting methylation, it is important to note that this method likely leads to the underestimation of methylation rate.

Using RNA sequence-based approaches, previous groups have identified variable methylation across 11 different p9 sites within mt-tRNAs of healthy Caucasian individuals; this methylation was discovered to be significantly associated with nuclear gene variants (i.e. *MRPP3*) [15]. In this study, we examined methylation at these same 11 mitochondrial p9 sites in the context of different neurodegenerative pathologies and identified associated nuclear genetic variants and gene expression. The results point to altered cellular processes that may be contributing to the mitochondrial dysfunction occurring within the cerebellum that is a hallmark of both Alzheimer’s disease (AD) and progressive supranuclear palsy (PSP).

## Methods

### Samples used

Samples used for this study were post-mortem cerebellar tissue of 266 elderly Caucasian individuals who were either healthy or diagnosed with Alzheimer’s disease (AD), progressive supranuclear palsy (PSP) or pathological aging (PA) (Table 1). Samples were originally collected by the Mayo RNAseq study led by Dr. Nilüfer Ertekin-Taner, Mayo Clinic, Jacksonville, FL as part of the multi-PI U01 AG046139 (MPIs Golde, Ertekin-Taner, Younkin, Price). All individuals with AD “…had definite diagnosis according to the NINCDS-ADRDA criteria and had Braak NFT stage of IV or greater. Control subjects had Braak NFT stage of III or less, CERAD neuritic and cortical plaque densities of 0 (none) or 1 (sparse) and lacked any of the following pathologic diagnoses: AD, Parkinson’s disease (PD), DLB, VaD, PSP, motor neuron disease (MND), CBD, Pick’s disease (PiD), Huntington’s disease (HD), FTLD, hippocampal sclerosis (HipScl) or dementia lacking distinctive histology (DLDH)” (doi:10.7303/syn5550404).

**Table 1:** Demographics of subjects used

Diagnosis	Sex	Age at Death	APOE		
			e2	e3	e4
NC (n = 74)	Male (*n* = 40)	80.93 ± 8.15	7.43%	86.40%	6.08%
	Female (*n* = 34)	85.15 ± 6.76			
AD (n = 82)	Male (n = 34)	81.68 ± 8.17	2.44%	68.30%	29.30%
	Female (*n* = 48)	83.21 ± 7.19			
PSP (n = 83)	Male (*n* = 51)	74.22 ± 6.94	9.04%	82.53%	8.43%
	Female (*n* = 32)	73.65 ± 5.99			
PA (n = 27)	Male (*n* = 11)	85.18 ± 3.74	7.41%	75.92%	16.67%
	Female (*n* = 16)	84.06 ± 4.65			

Age is reported as an average ± standard deviation for each respective group. Normal control (NC), Alzheimer’s disease (AD), progressive supranuclear palsy (PSP), pathological aging (PA).

### Data obtainment and QC

SNP, RNA-seq data and gene expression data were obtained from the Synapse Data Repository (doi:10.7303/syn5550404). Genomic and RNA-seq data was originally generated and QC’d by the Golde, Ertekin-Taner, Younkin and Price laboratories. Briefly, genotypes were obtained using the Illumina Human Omni 2.5Exome array. The protocol used for QC of genomic data included the following procedures: (1) duplicate or relatedness, (2) discrepant sex information, (3) low genotyping call rate (98%) and (4) outlying heterozygosity (± 3 standard deviations from the mean) were removed using PLINK. EIGENSTRAT was used to identify outliers (> 6 standard deviations from the mean after 5 iterations) [16]. Based on this QC protocol, a total of 10 individuals were removed from the original dataset of 278 individuals.

Briefly, RNA was extracted from brain tissue using TRIzol® and Qiagen RNeasy columns treated with DNase. Sequencing libraries were prepared using the Illumina Truseq kit. An Illumina Hiseq 2000 was used to obtain 101 bp paired-end reads (performed in triplicate; 3 lanes/sample). RNA-seq data of individuals with low mapped reads (< 85%) or sex discrepant gene counts (Y-chromosome gene expression) were removed from further analysis (20 individuals total). Using SNAPR, raw RNA-seq reads were aligned using the human genome and transcriptome builds GRCh38 and GRCh38.77 respectively, while filtering on Phred scores <= 20 [17]. Subsequent BAM files were utilized for determining alternative allele frequency at the 11 p9 sites. Gene expression was derived from transcript counts generated using SNAPR [17]; counts were normalized using the trimmed mean of m-values (TMM) method in edgeR taking into account differences in library size (calcNormFactors).

### Mitochondrial RNA heteroplasmy

The aim of this study was to investigate post-transcriptional mitochondrial p9 methylation in the context of neurodegenerative phenotypes. Mitochondrial specific reads were extracted from total RNA-seq BAM files using samtools v.1.34 *view.* An mpileup file was created from each BAM file using samtools *mpileup*. Heteroplasmy was measured at 11 different p9 sites in the mitochondrial transcriptome (585, 1610, 4271, 5520, 7526, 8303, 9999, 10,413, 12,146, 12,274, 14,734) from the mpileup files using *readcounts* in VarScan v.2.4 with default parameters. Of note, none of the aforementioned p9 sites have previously been shown to overlap with mitochondrial pseudogene (i.e. nuclear mitochondrial DNA (NUMT)) sequences [15]. Frequency of alternative allele calls (non-reference calls based on Cambridge Reference Sequence (CRS)) was measured at each of the 11 sites. Degree of post-transcriptional methylation was inferred from the frequency of alternative allele calls based on previous published methods [15]. A Kolmogorov-Smirnov test determined the data to have a non-normal distribution. Correlation coefficients for p9 methylation across all 11 sites were determined using Spearman’s rho. Degree of p9 methylation was analyzed between disease states using pairwise Kruskal-Wallis tests with Bonferroni correction for multiple testing at each individual site (not accounting for the 11 site tests).

### Gene-based genome-wide association

Gene-based association studies were utilized to identify nuclear encoded genes significantly associated with p9 methylation. In brief, principle component analysis using EIGENSOFT was conducted to determine if a considerable amount of heterogeneity was present in the SNP data [18]. Linkage disequilibrium-based pruning of the SNPs was conducted in PLINK (−-indep-pairwise 50 5 0.2) yielding a final dataset of 58,174 SNPs in linkage equilibrium [19]. Linear association adjusting for age, sex and eigenvectors 1–10 was applied to identify genetic variants associated with degree of methylation (log10 transformed) at each respective p9 site using the SNP2GENE function of the Functional Mapping and Annotation (FUMA) platform [20]; this utilizes MAGMA to map SNPs to protein-coding genes [21]. Position map window size was conservatively set to 10 kb. The genome-wide significance threshold was set to a Bonferroni adjusted *p*-value of 2.93 × 10^− 6^ based on the mapping of input SNPs to 17,079 genes; Bonferroni correction was applied within each linear association test. Manhattan plots and local regional plots as well as Q-Q plots to identify genomic inflation in the SNP-set used for the gene-based GWAS, were generated in FUMA. CADD scores reported for each gene are based on the top leading SNP/posMapMaxCADD as determined in FUMA.

### Gene expression analysis

To identify potential downstream effects (i.e. biological changes) linked to p9 methylation, we investigated nuclear gene expression significantly correlated with methylation. Correlations between nuclear genes and methylation at site 585 were determined using Spearman’s rho. The top 1000 transcripts (565 unique genes) and the first two principle components were visualized using the ClustVis online tool [22]. Rows and columns of the heatmap were clustered based on correlation distance and average linkage with unit variance scaling. The top genes (based on top 1000 transcripts) were input into the PANTHER classification system to identify overrepresented cellular components, molecular functions and/or biological processes using Fisher’s exact test with FDR correction [23]. For a more targeted enrichment analysis, all 14,306 transcripts (5300 unique genes) significantly correlated with p9 methylation were input into Ingenuity Pathway Analysis (IPA) (Qiagen Inc., https://www.qiagenbioinformatics.com/products/ingenuitypathway-analysis) and were tested for pathway enrichment using the Core Analysis function and a cerebellum specific reference.

## Results

### Mitochondrial tRNA methylation

Frequencies of alternative allele calls measured at 11 previously identified p9 sites (585, 1610, 4271, 5520, 7526, 8303, 9999, 10,413, 12,146, 12,274, 14,734) based on the revised Cambridge reference sequence, were used to infer degree of post-transcriptional methylation. High levels of mt-RNA sequence variation were observed across all individuals (Additional Figure 1). Degree of methylation, inferred from multi-allelic calls in RNA-seq data, was highly correlated across all 11 p9 sites with exception of 4271 and 14,734 (Additional Figure 2). For each p9 site, comparisons were made between each of the four groups: normal controls (NC) (*n* = 74), Alzheimer’s disease (AD) (*n* = 82), progressive supranuclear palsy (PSP) (*n* = 83) and pathological aging (PA) (*n* = 27) to assess differences in methylation levels. Non-parametric (Kruskal-Wallis) pairwise comparisons by disease state revealed significant differences in methylation at a number of p9 sites, with AD and PSP showing similar degrees of hypermethylation in comparison to NC and PA; no significant difference was seen between NC and PA (Table 2; Additional file 4). Site 585 was found to be the most variable, though the reasoning for this is unclear.

**Table 2:**
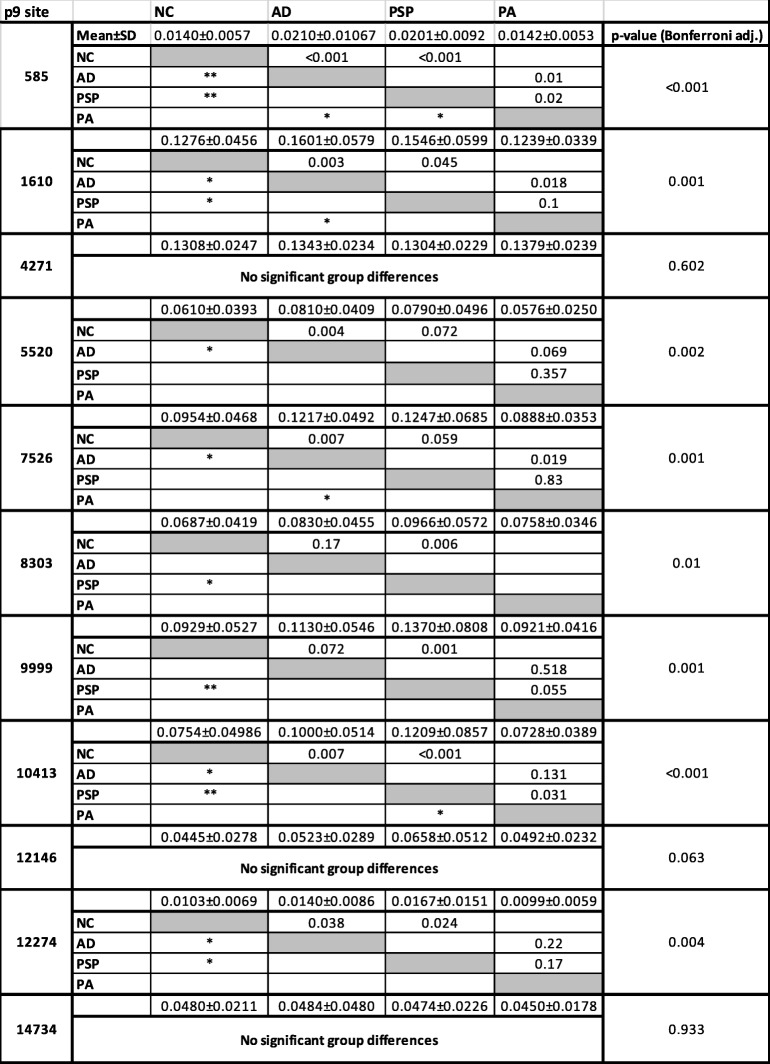
Summary table detailing p9 methylation data distributions and significant group differences per site

Mean ± standard deviation of degree of methylation (inferred from alternative allele frequency) is shown across the top for each site. Non-parametric Kruskal-Wallis *p*-values with Bonferroni correction (to adjust for multiple comparisons) is shown on the far right. *P*-values from pair-wise Kruskal-Wallis comparisons between groups are shown within the matrix; p-values< 0.05 (*), p-values< 0.001 (**).

### Nuclear gene-based associations

**Fig. 1:**
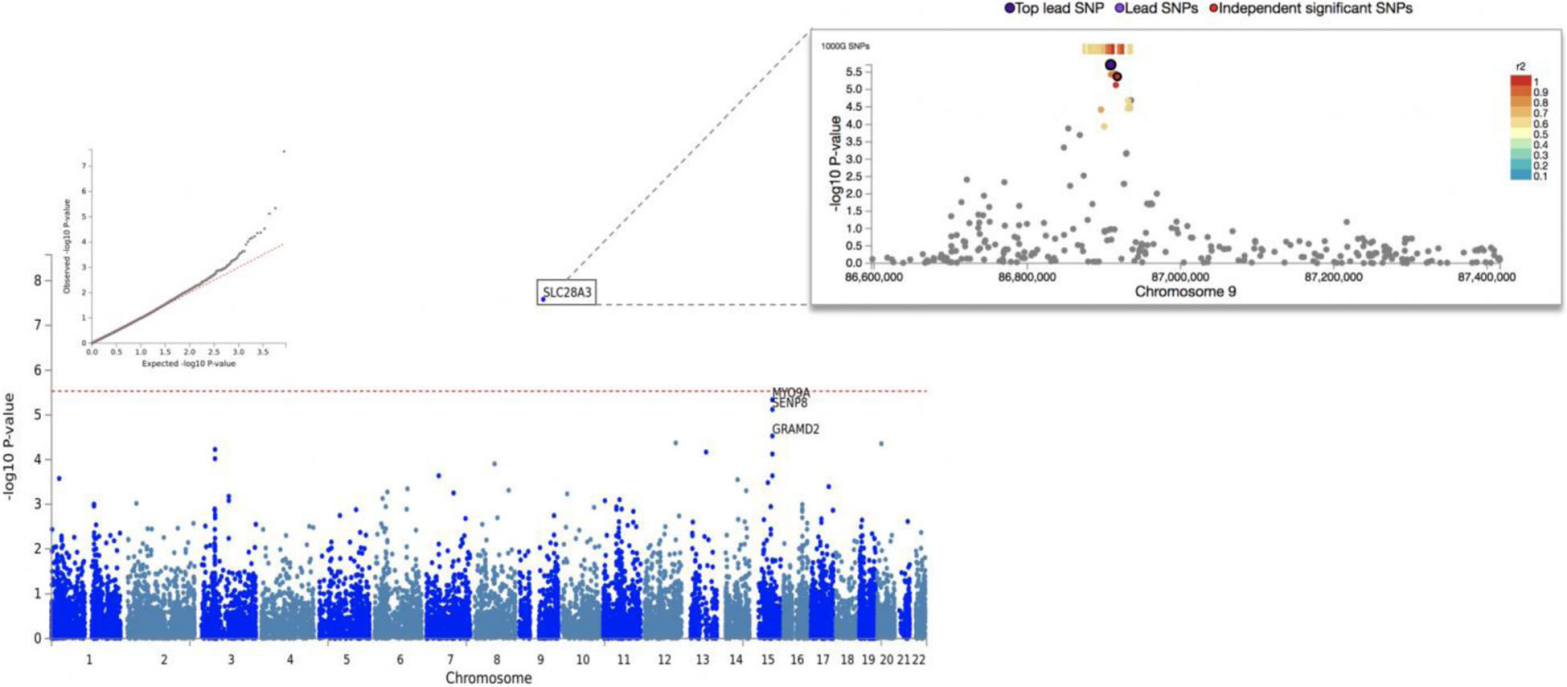
Association results for gene-based GWAS with methylation at site 585. The Q-Q plot (top left) conforms to expectations and shows no sign of genomic inflation. The Manhattan plot (bottom left) illustrates a primary signal in *SLC28A3* on chromosome 9 (p-value = 1.935 × 10^− 6^); as well as multiple suggestive associations on chromosome 15. The regional plot (top right) displays clustering of individual SNPs within an 800 kb window and shows multiple SNPs in LD with the top lead SNP in the gene-based signal

**Fig. 2:**
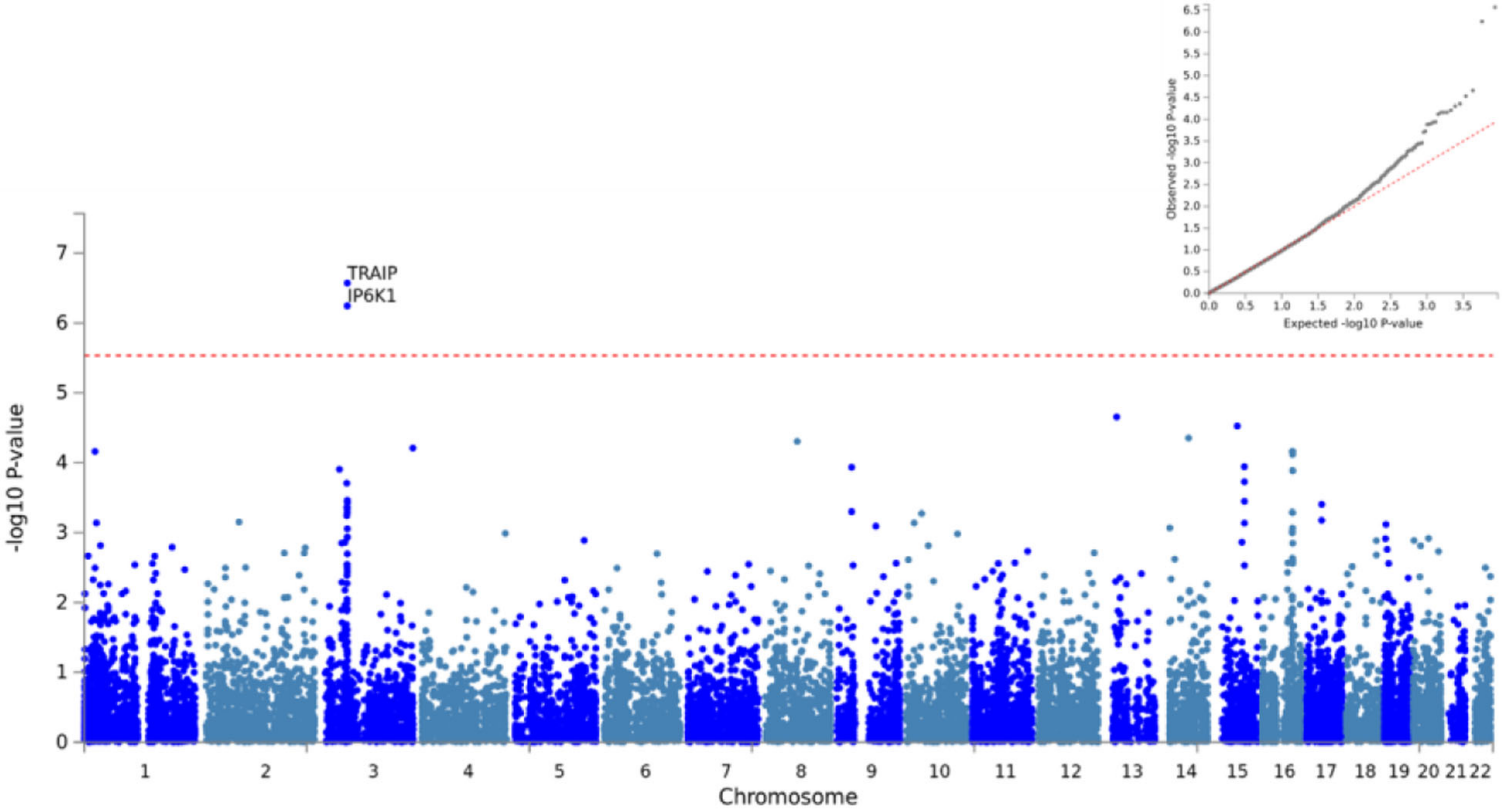
Association results for gene-based GWAS with methylation across 6 out of 11 sites. The Q-Q plot (top right) conforms to expectations and shows no sign of genomic inflation. The Manhattan plot (bottom left) illustrates a primary signal in *TRAIP* and *IP6K1* on chromosome 3 (8.32 × 10^− 7^, 6.67 × 10^− 7^). This result was replicated in 6 out of 11 p9 sites (results for site 12,146 shown here)

Gene-based GWAS mapped typed SNPs to 17,079 protein coding genes. Two SNPs (rs12343928; rs4877837) located on chromosome 9 were mapped to *SLC28A3* (p-value = 1.935 × 10^− 6^, CADD score = 24.7) and were determined to be significantly associated with methylation at site 585; the regional plot reveals that several SNPs in LD with the top lead SNP are also associated in the local signal (Fig. 1; Additional file 2). A single SNP (rs2034879) mapping to *SENP8* (CADD score = 13.22)*, MYO9A* (CADD score = 18.35)*, GRAMD2* (CADD score = 16.49) on chromosome 15 had suggestive associations with methylation at p9 site 585 (Fig. 1; Additional file 2). Another SNP (rs9872864) mapping to *TRAIP* (*p*-value = 8.32 × 10^− 7^, CADD score = 7.558) and nearby gene *IP6K1* (p-value = 6.67 × 10^− 7^, CADD score = 20.7) located on chromosome 3 was also found to have significant associations with methylation at six out of the 11 p9 sites (5520, 7526, 8303, 9999, 10,413, 12,146) (Fig. 2; Additional file 2).

### Mitochondrial-related gene expression

**Fig. 3:**
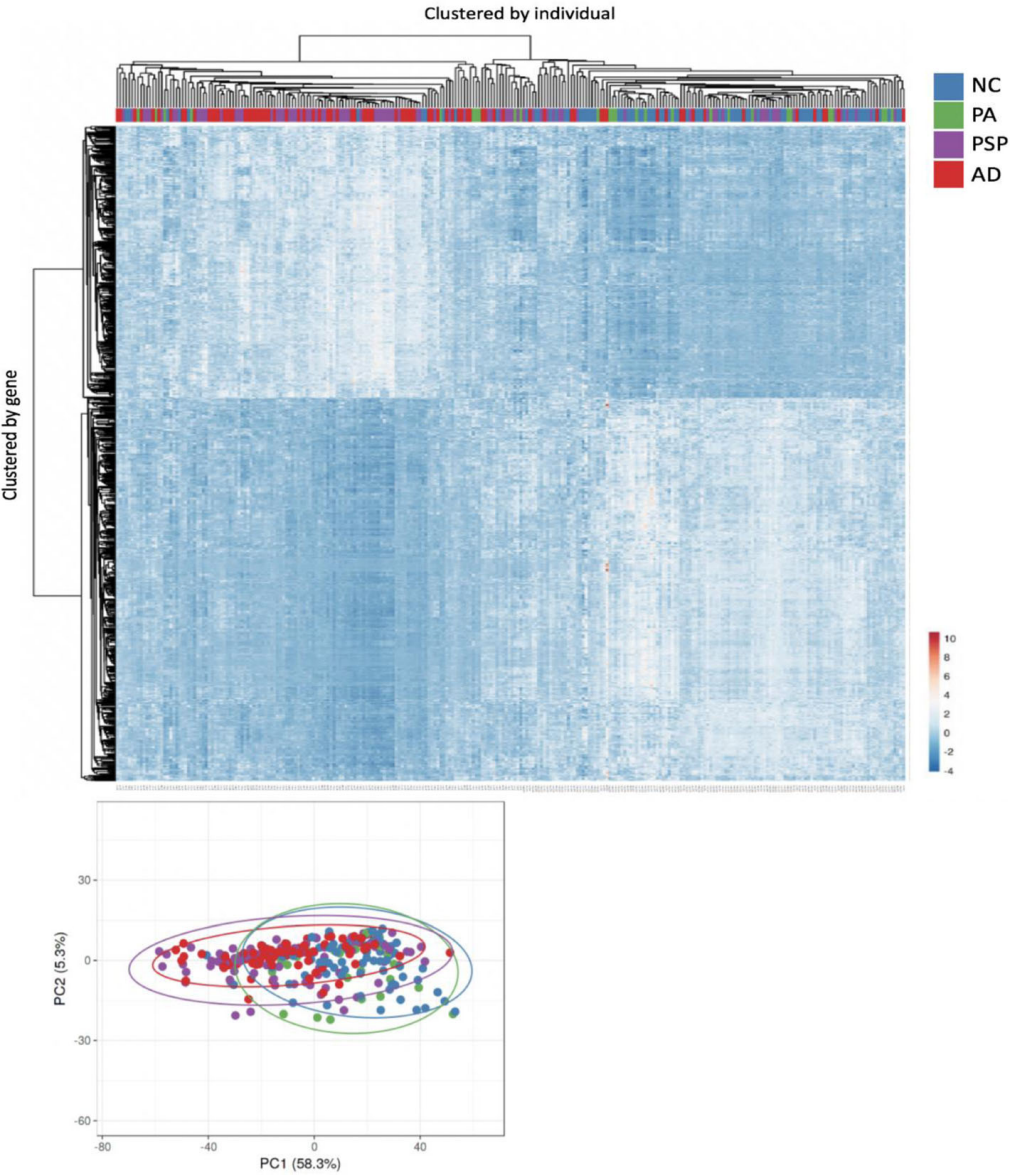
Heatmap displaying the top 1000 transcripts significantly associated with degree of p9 methylation at site 585. Expression profiles are clustered by gene (y-axis) and individual (x-axis) (top). Principle component analysis of gene expression displays overlap between case groups (bottom left). Colors represent disease status; NC (blue), PA (green), AD (red) and PSP (purple) (bottom). Gradient bar (bottom right) represents gene expression level; high expression (red), low expression (blue)

Expression of 5300 transcripts was found to significantly correlate with degree of methylation at site 585; the top 1000 transcripts (565 unique genes) are visualized in Fig. 3. Based on the primary bifurcation, left and right clades appear to be enriched for AD and PSP, and NC and PA respectively; though enrichment was not formally tested (Fig. 3).

**Fig. 4:**
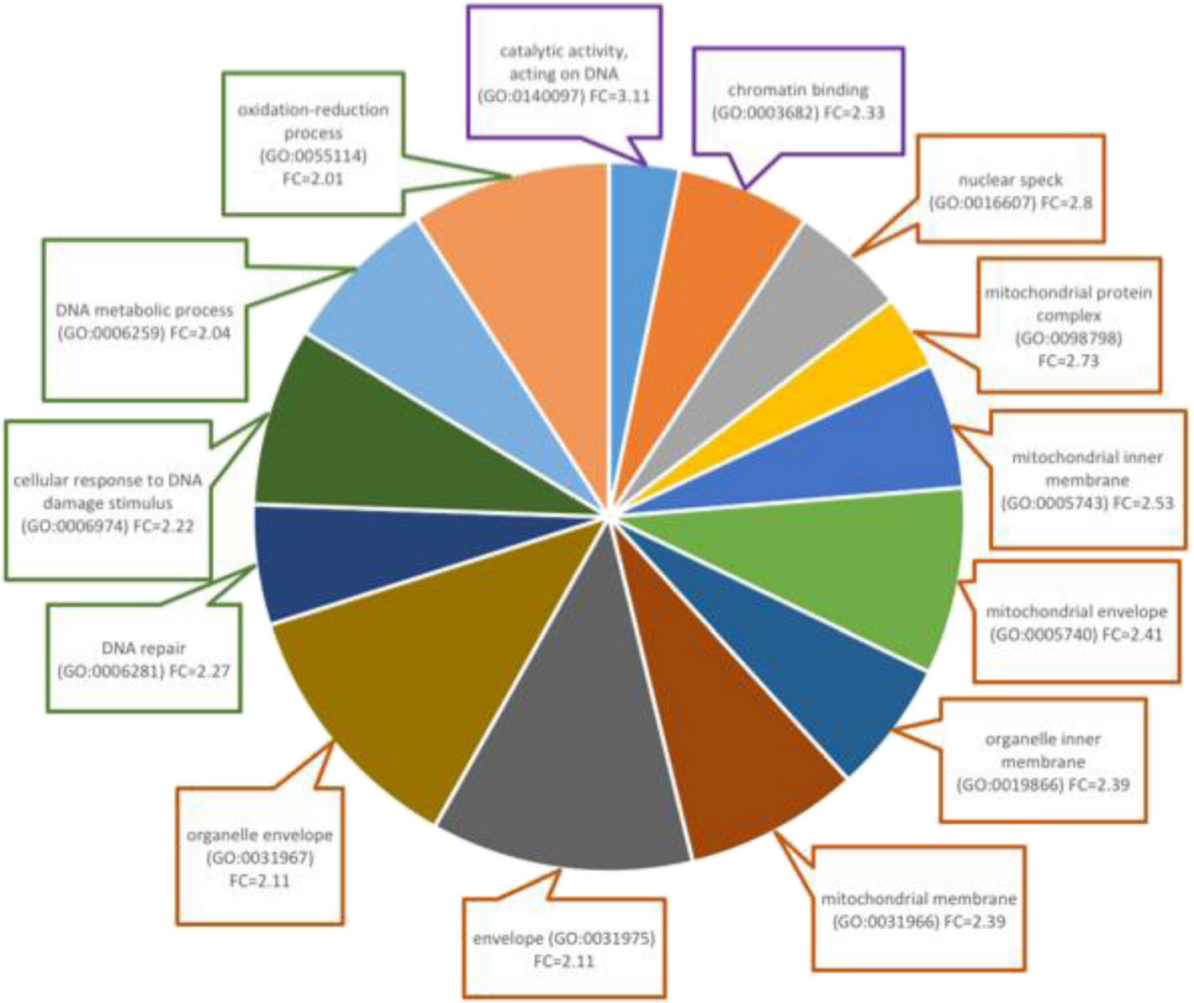
Overrepresented GO terms based on the top 1000 transcripts significantly correlated with p9 methylation. GO terms are color coded by category; Molecular function (purple), cellular component (orange), biological process (green). Terms were selected using a threshold of > 2 fold change. Fold change was determined based on number of enriched genes from our 1000 transcripts list divided by the number of expected enriched genes

The top 1000 transcripts correlated with p9 methylation at site 585 were analyzed using PANTHER to test for significantly overrepresented cellular components, molecular functions and biological processes. We identified a number of GO terms to be overrepresented in our gene-set including several that were mitochondrially-associated (Fig. 4; Additional file 3).

**Fig. 5:**
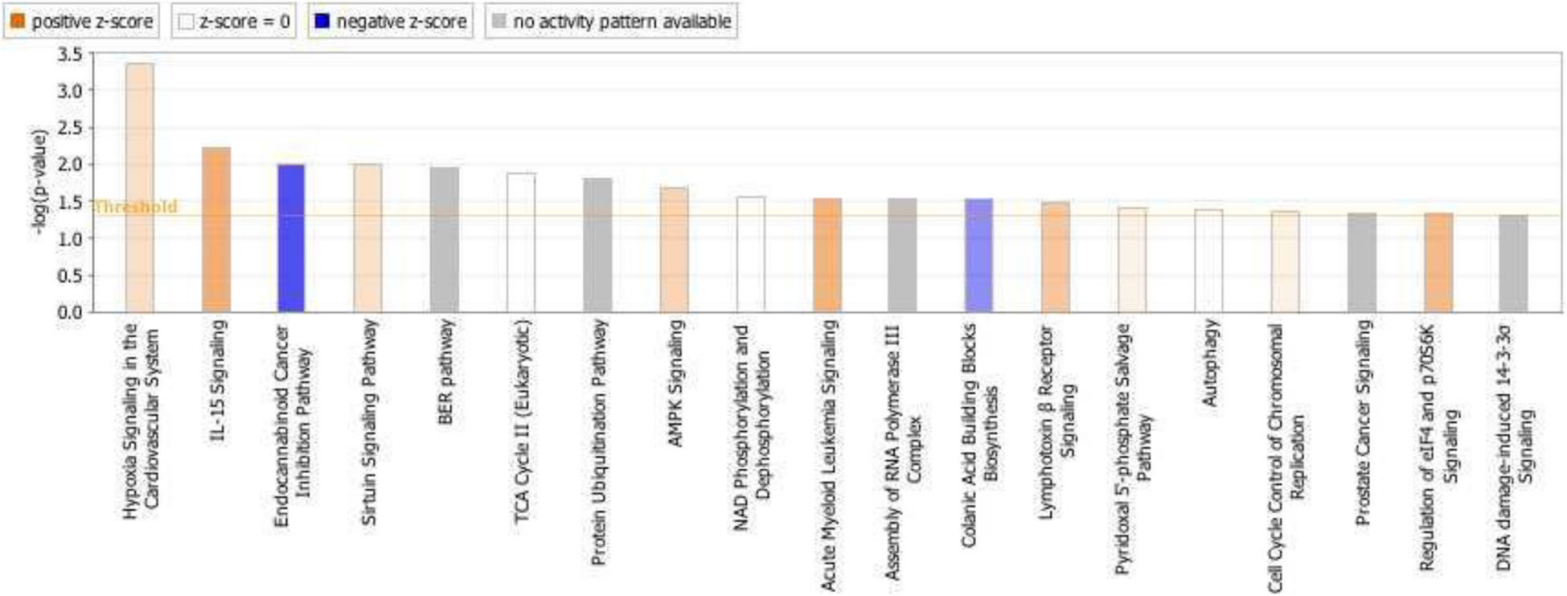
Enriched canonical pathways based on all transcripts significantly correlated with p9 methylation. Pathways positively and negatively correlated with p9 methylation are shown in orange and blue respectively

A tissue-specific (i.e. cerebellum) enrichment analysis was then conducted in IPA using all significantly correlated transcripts (5300 unique genes). Several pathways were identified as enriched in our gene-set including many that are linked to mitochondrial function as well as DNA damage/repair (Fig. 5).

## Discussion

Our analysis of RNA sequence has validated that methylation occurs consistently at functionally important sites across the mitochondrial transcriptome, and that this methylation is highly correlated across the 11 multiallelic sites. Here we show significant hypermethylation at specific sites in the mitochondrial transcriptome (i.e. mt-tRNA p9 sites) in the cerebellar tissue of individuals with Alzheimer’s disease and progressive supranuclear palsy. Similarities in methylation between these two pathologies may suggest a comparable genetic etiology underlying the cerebellar mitochondrial dysfunction that is a hallmark of both diseases [24], [25]. Interestingly, no significant difference was observed between NC and PA, which may be due to the ambiguity of differentiating healthy from pathological aging. Individuals diagnosed with pathological aging often appear to be cognitively intact despite the presence of neuritic plaques. Similar presentation has also been observed in healthy aged individuals, making it difficult to distinguish between the two diagnoses [26]. Moreover, a recent study by Idaghdour and Hodgkinson (2017) revealed significant hypermethylation at mitochondrial p9 sites in cancer. This substantiates the notion that mt-tRNA hypermethylation may be more closely associated with pathology rather than normal aging processes. Whether this hypermethylation is a cause or consequence of pathology is still unclear.

Nevertheless, it is important to acknowledge the technical limitations associated with traditional RNA sequencing workflows. Here we sought to investigate post-transcriptional methylation of mt-tRNAs using total RNA sequence data. Importantly, these RNA molecules are highly folded and small in nature, often making them difficult to capture. Traditional total RNA sequencing protocols, such as that used here often 1) do not enrich for, and 2) usually detail removal of, small RNAs. This means that mature mt-tRNA reads may have been under-represented in this dataset. Therefore, any conclusions made here may not be based on the entire mature mt-tRNA pool. Future studies investigating p9 methylation should aim to use small RNA enrichment protocols, as well as perform cDNA size selection prior to RNA sequencing.

### Nuclear genes are associated with p9 methylation

The gene-based genome-wide association analysis revealed that variation in several nuclear encoded genes is significantly associated with methylation at different p9 sites throughout the mitochondrial transcriptome. Two SNPs (rs12343928; rs4877837) mapping to solute carrier family 28 member 3 (*SLC28A3*) located on chromosome 9 were found to be significantly associated with methylation at p9 site 585. The strength of this result was further substantiated by the clustering of neighbouring SNPs in linkage disequilibrium within the gene region (Fig. 1). The protein encoded by this gene is involved in regulating neurotransmission, vascular tone and metabolism of nucleoside drugs, which are processes that are physiologically relevant to our tissue and diseases of interest. Nucleoside drugs are often used to treat age-related diseases such as type 2 diabetes and hypertension, as well as mitochondrial dysfunction in Parkinson’s disease (PD) [27]. This is interesting, as PSP pathology also features mitochondrial dysfunction and bears phenotypic similarity to PD, while T2D and hypertension often serve as comorbidities for AD.

A single SNP (rs2034879) that mapped to several genes (*SENP8, MYO9A, GRAMD2*) on chromosome 15 (likely due to the restrictive window size of ±10 kb that was used) reached the suggestive threshold for association with p9 methylation at position 585. SENP8 is known to catalyze pathways associated with neddylation, a post-transcriptional modification analogous to ubiquitination; these pathways are also responsible for the endocytic degradation of APP. Dysfunction of these pathways has been observed in AD leading to characteristic accumulation of APP and Aβ [28]. MYO9A is an unconventional myosin responsible for the regulation of Rho GTPase activity within neurons, which in turn regulates neuronal morphology and function; for these reasons, the Rho family has been a recent therapeutic target for neurodegenerative diseases such as AD [29]. In turn, various Rho GTPases have also been implicated in maintenance of mitochondrial homeostasis and apoptotic signaling [30]. GRAMD2 is involved in the organization of endoplasmic reticulum-plasma membrane contact sites (EPCS), which are key modulators of calcium homeostasis [31]. Calcium serves as an important regulator of mitochondrial bioenergetics (e.g. activation of different Kreb’s cycle enzymes [32]), dynamics and apoptotic signaling. Subsequent contact sites between the ER and mitochondria, termed mitochondrial associated membranes (MAMs), facilitate uptake of calcium from the ER to the mitochondria [33], although there is not a clear role for GRAMD2 in this process.

Methylation at six of the 11 p9 sites (5520, 7526, 8303, 9999, 10,413, 12,146) was significantly associated with a single SNP (rs9872864) mapping to two genes *TRAIP* and *IP6K1* located on chromosome 3. *TRAIP* encodes TRAF interacting protein, an E3 ubiquitin ligase that plays a key role in cell survival and apoptosis. TRAIP serves important roles in cell cycle checkpoints by regulating spindle assembly, appropriate chromosome distribution during cell division, and DNA damage responses [34]. Cell cycle checkpoints have been proposed to serve neuroprotective roles; checkpoint dysregulation and cell cycle re-entry of post-mitotic neurons have been observed in a number of tauopathies [35]. *IP6K1* encodes an inositol phosphokinase responsible for synthesis of 5-diphosphoinositol phentakisphosphate (5-IP_7_) from hexakisphosphate (IP_6_). IP6K1 serves as an important upstream regulator for various metabolic processes (e.g. glucose homeostasis, lipolysis), apoptosis and global transcription [36]. It has been investigated as a therapeutic target for obesity and type 2 diabetes [37] and variants within the *IP6K1* gene region have been previously identified, using tag SNP analysis, as being associated with AD [38]. IP6K1 also impacts mitochondrial function by regulating ATP concentration via alteration of the ratio of glycolytic to oxidative phosphorylation [39]. Knockout (*IP6K1*−/−) yeast models have shown decreased mitochondrial respiration, yet increases in ATP, a paradox that is thought to be due to enhanced glycolysis and depletion of metabolic processes requiring ATP [39]. Further, its role in lipolysis is pathologically relevant as findings from Wan et al. [40] demonstrate the capacity of Aβ accumulation to promote lipolysis, increasing lipid toxicity and lipid peroxidation, leading to subsequent downstream mitochondrial dysfunction [41], a phenotype common to many neurodegenerative diseases.

While several of the genes associated here are loosely connected to mitochondrial function, we did not identify any loci that may directly affect mt-tRNA processing. It is interesting to note that 5 out of the 6 genes identified in the gene-based GWAS had CADD scores > 10 (based on the top leading SNP or maximum CADD score of variants within the gene region). In brief, Combined Annotation Dependent Depletion (CADD) scoring is a predictive machine learning tool that allows for estimation of the deleteriousness and/or pathogenicity of causal genetic variants [42]. In general, raw CADD scores exceeding 10 are considered to be in the top 10% of deleterious variants in the human genome [42]. Our findings here suggest that the genes identified as having significant associations with p9 methylation, may play a role in pathogenesis; though their exact role remains ambiguous at this time.

Hodkinson et al. [15], reported p9 methylation associations with several genes—one of which is *MRPP3*, a key player in tRNA processing in the mitochondria. We did not replicate the *MRPP3* association which is not entirely surprising since their study was conducted in normal adults (40–69 years) without any particular pathological conditions versus our study of aged subjects with neurodegenerative disease. It may follow that the variability in *MRPP3* associated with p9 methylation levels described in Hodgkinson et al., is presumably normal, whereas p9 methylation in our cohort is presumably a result of pathological process(es). The resulting genotype associations may be pointing to novel gene variants that are upstream effectors of mitochondrial function. Conversely, it is important to acknowledge the caveat that gene-based association testing was performed together on all individuals of this cohort; the small sample size of each diagnostic category (NC, AD, PSP, PA) left us underpowered to detect significant associations when analyzing by each group separately. Given that we identified p9 hypermethylation in both AD and PSP, it is possible that genes identified here as significantly associated with p9 methylation may be related to individual pathologies and not necessarily post-transcriptional RNA processes.

### Mitochondrially-related gene expression is correlated with p9 methylation

We identified 5300 genes significantly correlated with p9 methylation. Using principle component analysis, we identified clustering of gene expression profiles by disease state with AD and PSP, and NC and PA grouping together respectively. Of the top gene expression hits, we identified over-represented GO terms related to a number of cellular components and processes using PANTHER. Interestingly, there was over-representation of mitochondrial components.

In order to identify tissue-specific changes, we performed further enrichment analysis in IPA using a cerebellum tissue reference and all of the transcripts (5300 unique genes) significantly correlated with p9 methylation. Again, several of the enriched pathways identified were linked to mitochondrial function and homeostasis, as well as DNA damage/repair. We also identified a number of pathways related to RNA processing. In addition, some of the GO terms (e.g. *DNA repair* and *cellular response to DNA damage stimulus*) and canonical pathways (e.g. *Cell cycle control of chromosomal replication*, *DNA damage induced 14–3-3σ signaling*) appeared to correlate with functions of one of the top genes (i.e.*TRAIP*) identified in the gene-based GWAS. Though some of the GO terms and canonical pathways are rather ambiguous, it does imply that p9 methylation is associated with altered transcription of gene-sets important for mitochondrial function.

## Conclusion

This is the first work analyzing mitochondrial p9 methylation in the context of neurodegeneration. Here we report an association between p9 methylation and nuclear-encoded gene expression, as well as mitochondrial-related regulation; although, the implications of these associations are unclear. We observed comparable post-transcriptional mt-tRNA hypermethylation and nuclear gene expression profiles in the cerebellum of Alzheimer’s disease and progressive supranuclear palsy patients. Although technical limitations in this analysis do not allow us to conclude on methylation status of the mature tRNA pool, the results here are likely indicative of molecular similarities underlying the mitochondrial dysfunction already known to occur within the cerebellum of individuals diagnosed with either of these tauopathies.

**Fig. 6:**
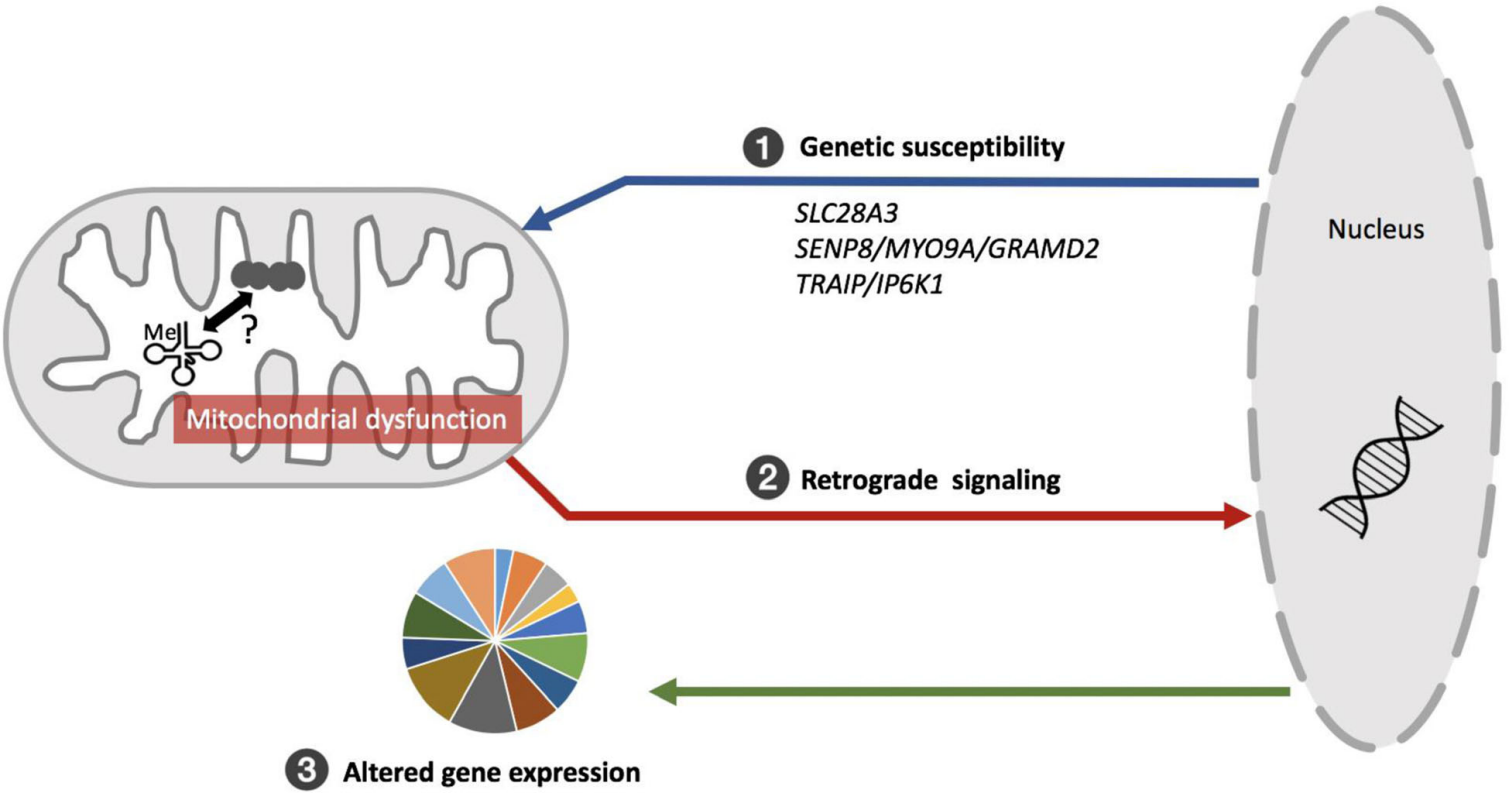
Hypothetical schematic of potential causes and effects of p9 methylation in neurodegeneration. (1) Nuclear encoded genetic variants may be causal for p9 hypermethylation within neuronal mitochondria, resulting in onset or exacerbation of electron transport chain dysfunction. (2) Subsequent mitochondrial dysfunction may lead to altered downstream nuclear gene expression via retrograde signaling resulting in (3) changes to various molecular and cellular processes that further contribute to neurodegenerative pathology

A basal level of p9 methylation is required for proper tRNA folding and stability [7] and the degree of methylation at p9 sites has been shown by other groups to have low inter-individual variability [43]. In addition, p9 hypermethylation has been observed in other age-related diseases such as cancer [44]. We propose that p9 hypermethylation in AD and PSP may be more closely associated with pathology as opposed to normal aging. As shown in Fig. 6, we observed that specific nuclear encoded variants associated with hypermethylation at mitochondrial p9 sites within cerebellar neurons, importantly causality is not clear (i.e. associations may be linked to pathological processes and not necessarily RNA methylation). For example, hypermethylation of p9 may be the primary event that impacts the function of the electron transport chain (ETC) and results in mitochondrial dysfunction, due to the influence that p9 methylation has on downstream mitochondrial protein translation. Alternatively, it is possible that ETC dysfunction is the primary event that causes altered p9 methylation, in which case, the p9 hypermethylation is serving as an endophenotype of genetic risk for mitochondrial dysfunction. Regardless, through retrograde signaling, mitochondrial dysfunction may impact nuclear gene expression resulting in alterations in cellular and molecular processes to further exacerbate neurodegenerative pathology.

Though at present, it is unclear whether mt-tRNA methylation is a cause or consequence of pathology, these results point to a potential role for mitochondrial post-transcriptional methylation in the pathophysiology of common tauopathies such as Alzheimer’s disease and progressive supranuclear palsy. Given this, post-transcriptional mt-tRNA methylation may serve as an exciting area of investigation for the development of future treatment strategies for neurodegenerative disease.

## Supplementary information

**Additional file 1.** Additional figures.

**Additional file 2.** Gene-based GWAS results.

**Additional file 3.** PANTHER over-representation test results.

**Additional file 4.** Boxplot by diagnosis.

## Data Availability

Data used in this study was obtained from the Synapse data repository (https://www.synapse.org/#!Synapse:syn5550404). Subsequent analyses not included in the additional materials are available by the author upon request.

## Abbreviations

mtDNA: Mitochondrial DNA
mt-RN: Mitochondrial RNA
mt-tRNA: Mitochondrial tRNA
p9: Position nine
NC: Normal control
AD: Alzheimer’s disease
PSP: Progressive supranuclear palsy
PA: Pathological aging
GWA: Genome-wide association study
SNP: Single nucleotide polymorphism
FDR: False discovery rate
CADD: Combined Annotation Dependent Depletion
NUMT: Nuclear mitochondrial DNA
ETC: Electron transport chain
EPCS: Endoplasmic reticulum-plasma membrane contact sites
MAMs: Mitochondrial associated membranes
TMM: Trimmed mean of m-value
