# Supplementary material for: Mitochondrial tRNA methylation in Alzheimer’s disease and progressive supranuclear palsy
Source: BMC Med Genomics. 2020 May 19;13:71. doi: 10.1186/s12920-020-0727-9 (PMC7236490; doi:10.1186/s12920-020-0727-9)
Supplement: Supplementary file 1 — Additional file 1. Additional figures. [file 12920_2020_727_MOESM1_ESM.docx]

**Additional Figures**

**
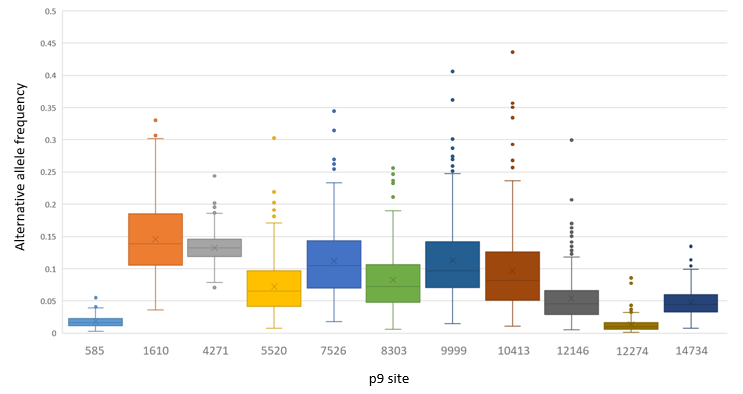
Additional Figure 1.** Variation in frequency of alternative allele at each of the 11 p9 sites. Alternative allele frequency is cumulative for all 266 individuals.

**
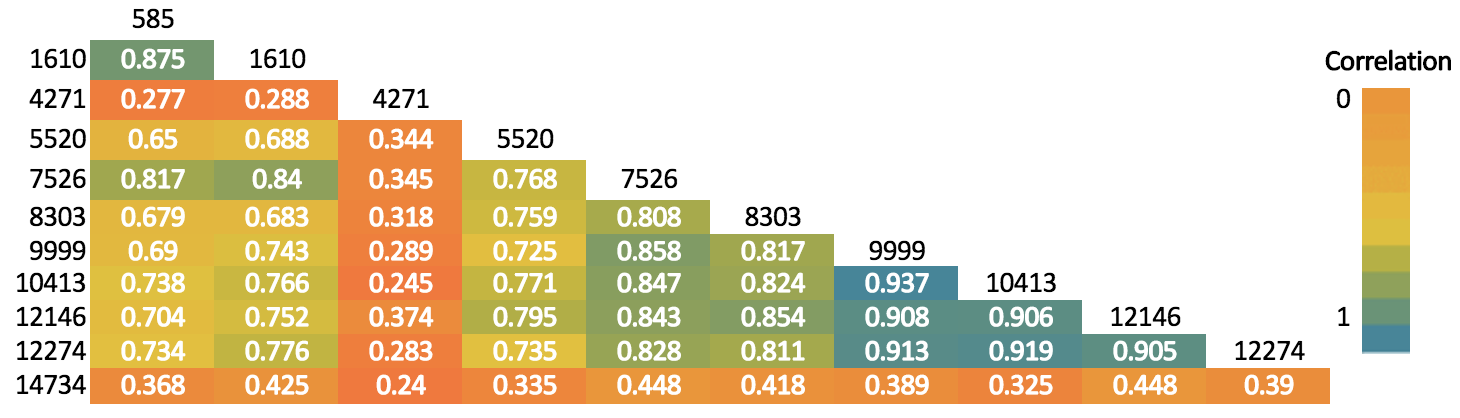
Additional Figure 2.** Matrix displaying Spearman Rho correlation of methylation across the 11 p9 analyzed sites.
