# Supplementary figures and images for: Mitochondrial tRNA methylation in Alzheimer’s disease and progressive supranuclear palsy
Source: BMC Med Genomics. 2020 May 19;13:71. doi: 10.1186/s12920-020-0727-9 (PMC7236490; doi:10.1186/s12920-020-0727-9)

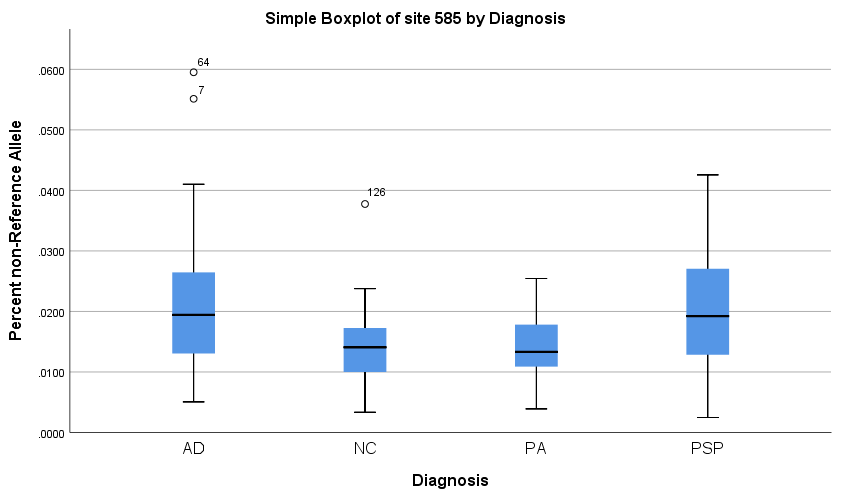


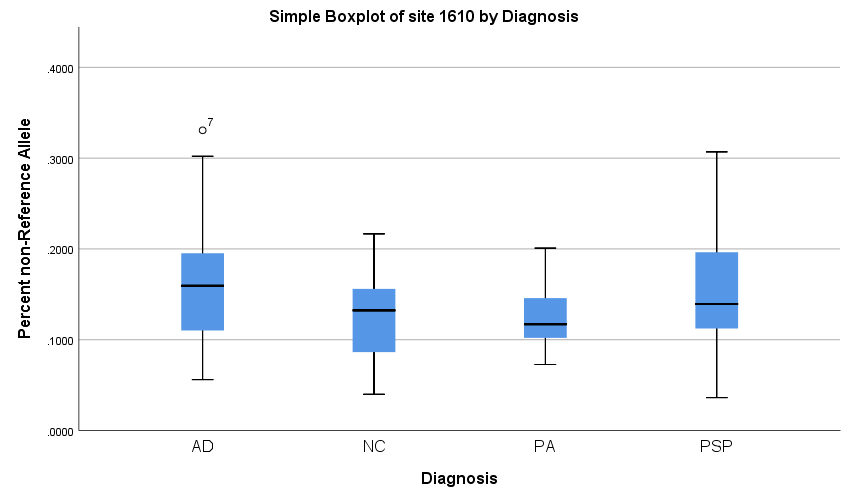


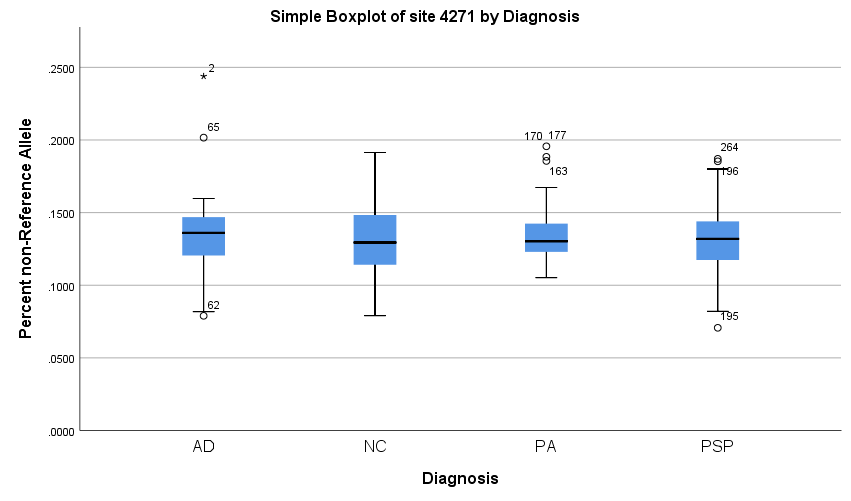


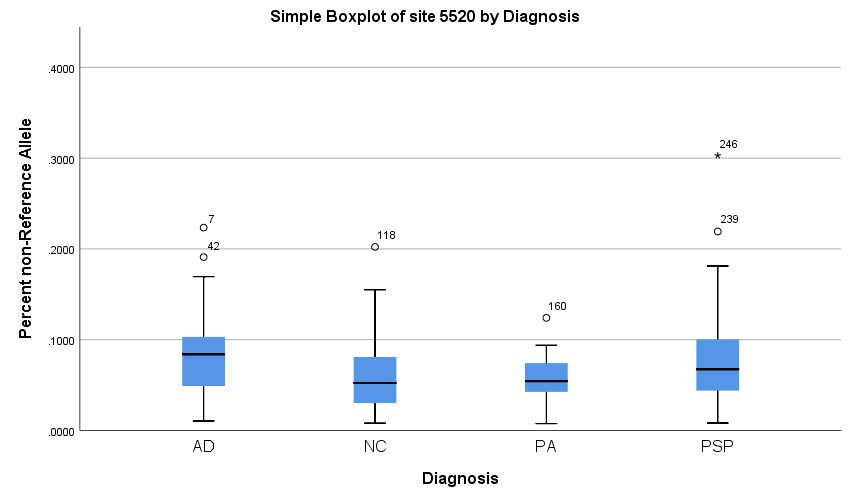


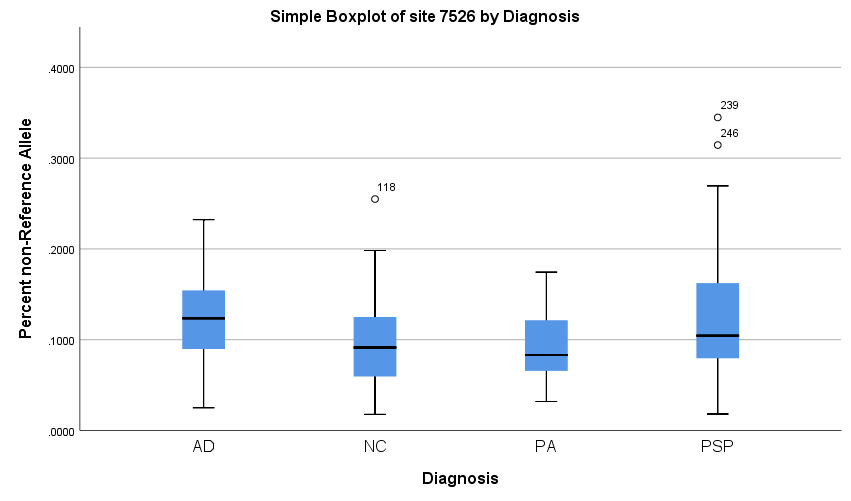


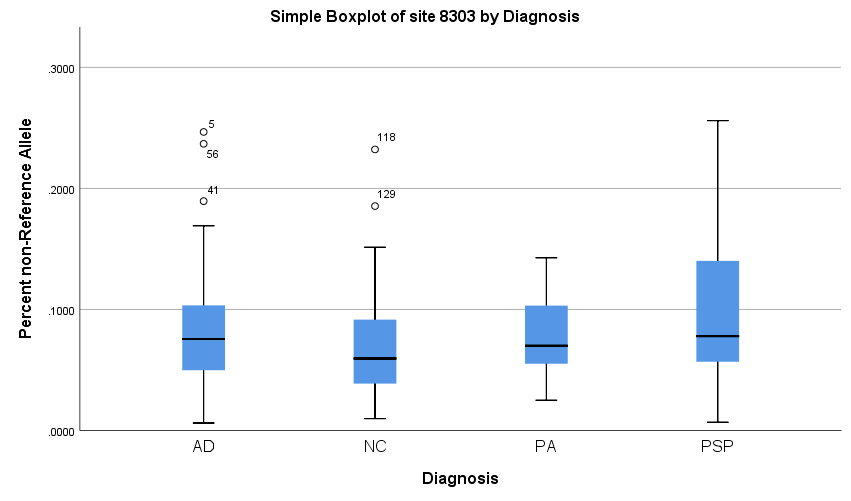


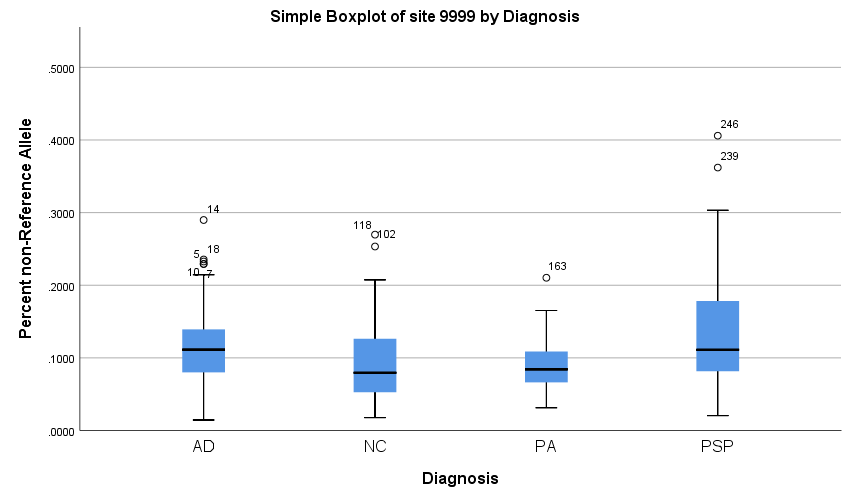


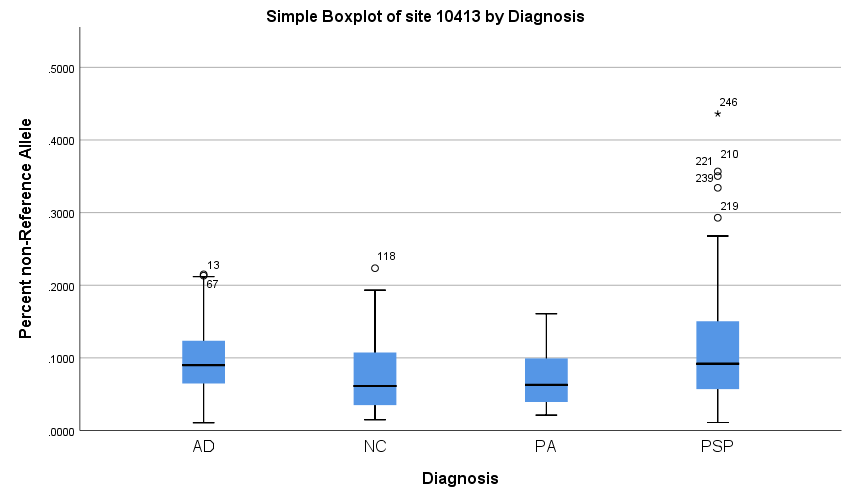


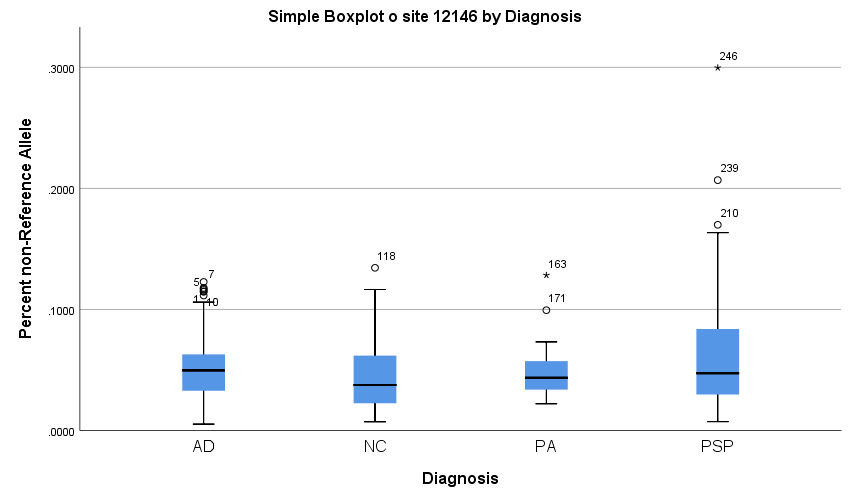


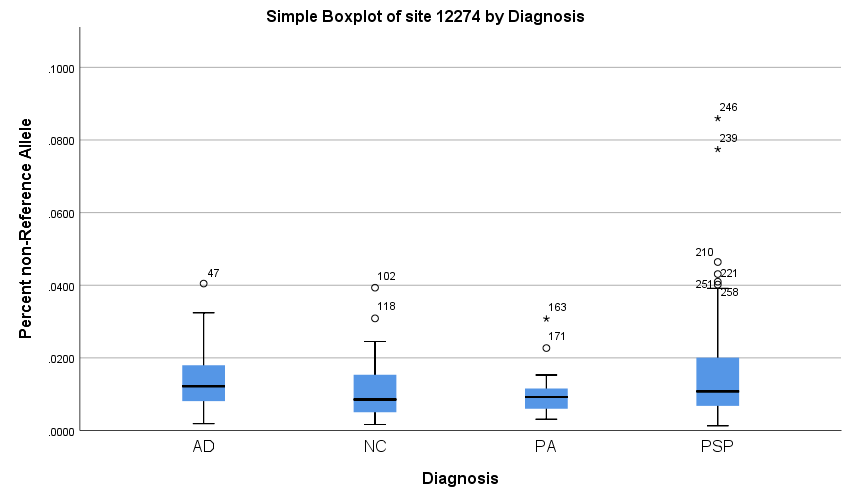


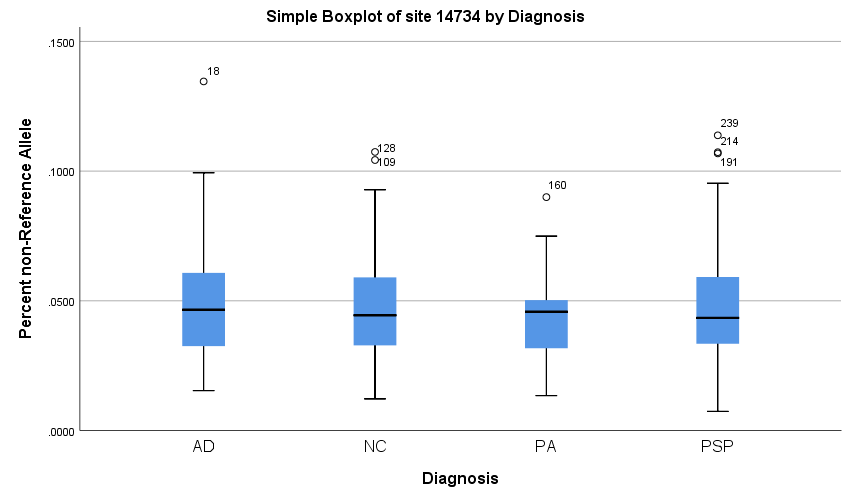

Supplement: Supplementary file 4 — Additional file 4. Boxplot by diagnosis. [file 12920_2020_727_MOESM4_ESM.docx]
